# Machine learning-empowered sleep staging classification using multi-modality signals

**DOI:** 10.1186/s12911-024-02522-2

**Published:** 2024-05-06

**Authors:** Santosh Kumar Satapathy, Biswajit Brahma, Baidyanath Panda, Paolo Barsocchi, Akash Kumar Bhoi

**Affiliations:** 1 Department of Information and Communication Technology, Pandit Deendayal Energy University, Gandhinagar, Gujarat 382007 India; 2 McKesson Corporation, 1 Post St, San Francisco, CA 94104 USA; 3 LTIMindtree, 1 American Row, 3Rd Floor, Hartford, CT 06103 USA; 4 Institute of Information Science and Technologies, National Research Council, 56124 Pisa, Italy; 5 Directorate of Research, Sikkim Manipal University, Gangtok, 737102 Sikkim India

**Keywords:** Polysomnography signals, Multi-modal analysis, Sleep staging, AASM rules, Machine learning, Random forest, Epoch-wise analysis

## Abstract

The goal is to enhance an automated sleep staging system's performance by leveraging the diverse signals captured through multi-modal polysomnography recordings. Three modalities of PSG signals, namely electroencephalogram (EEG), electrooculogram (EOG), and electromyogram (EMG), were considered to obtain the optimal fusions of the PSG signals, where 63 features were extracted. These include frequency-based, time-based, statistical-based, entropy-based, and non-linear-based features. We adopted the ReliefF (ReF) feature selection algorithms to find the suitable parts for each signal and superposition of PSG signals. Twelve top features were selected while correlated with the extracted feature sets' sleep stages. The selected features were fed into the AdaBoost with Random Forest (ADB + RF) classifier to validate the chosen segments and classify the sleep stages. This study's experiments were investigated by obtaining two testing schemes: epoch-wise testing and subject-wise testing. The suggested research was conducted using three publicly available datasets: ISRUC-Sleep subgroup1 (ISRUC-SG1), sleep-EDF(S-EDF), Physio bank CAP sleep database (PB-CAPSDB), and S-EDF-78 respectively. This work demonstrated that the proposed fusion strategy overestimates the common individual usage of PSG signals.

## Introduction

Sleep is a fundamental necessity for humans, crucial for maintaining physical and mental well-being [1]. Inadequate sleep patterns have been observed to lead to difficulties in learning, concentration, and decision-making and can impact social interactions. Prolonged adherence to such sleep behaviors may result in various sleep disorders. Notably, certain sleep disorders, like obstructive sleep apnea (OSA) [2], have direct or indirect associations with chronic diseases, such as an increased risk of stroke [3]. Additionally, insomnia has been linked to conditions like diabetes and cardiovascular diseases [4]. Therefore, assessing sleep quality and employing proper diagnostic procedures to address diverse sleep issues for overall health is imperative. Two main standards, R&K and AASM guidelines, examine sleep patterns and their attributes. R&K rules categorize the entire sleep cycle into seven stages, including Wake (W), Stage1 (S1), Stage2 (S2), Stage3 (S3), Stage4 (S4), Rapid Eye Movement (REM), and movement time. Stages S1 to S4 are considered non-REM sleep stages. In later research, the American Academy of Sleep Medicine (AASM) introduced updated guidelines, consolidating the sleep cycle into five stages: Wakefulness (W), N1, N2, and N3, with changes reflecting the measurement and treatment of S3 and S4 as part of the N3 stage [5].

**Fig. 1:**
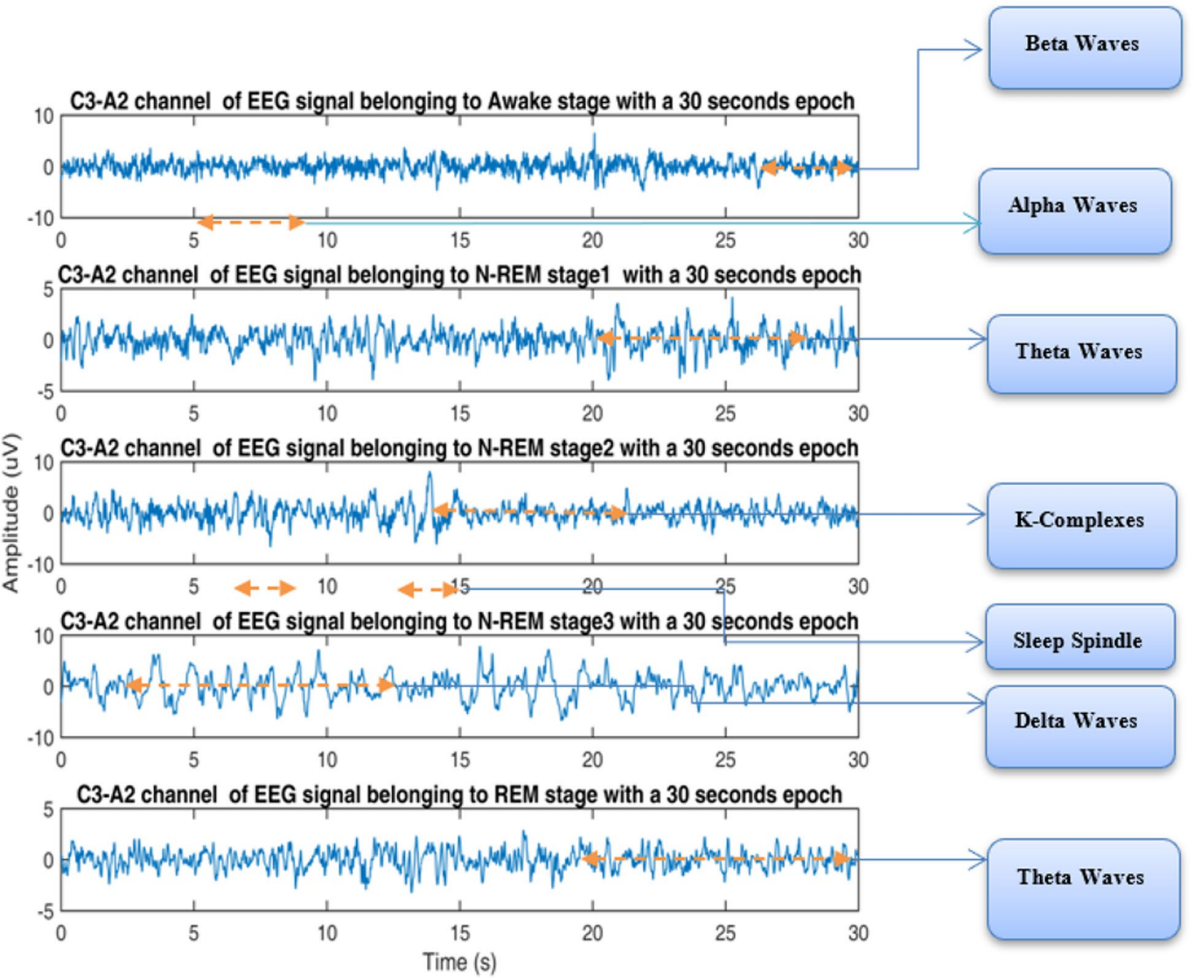
EEG patterns with the different sleep stages

Experts commonly employ the Polysomnography (PSG) test to assess different types of sleep disorders in subjects. PSG signals typically include an electroencephalogram (EEG) [6], electrocardiogram (ECG) [6], electrooculogram (EOG) [7], and electromyogram (EMG) [8]. These signals are recorded and analyzed visually by experts. The process involves at least two experts, one interpreting the signal waveforms while the other annotating them [9]. In the traditional diagnostic approach, manual inspection is used to observe and label the subject's sleep behavior. However, this method often yields lower performance due to variations in labeling and annotation skills among experts [10]. Additionally, reaching a consensus on sleep stage labels between the two experts can be challenging. As a result, many automated sleep staging systems have been developed to analyze sleep stages based on various sleep disorders, aiming to automate the scoring of sleep stages [11]. Figure 1 illustrates the EEG pattern of sleep stages. The depicted sleep EEG behavior is from subject id-61, a 61-year-old male, sourced from the Physio Bank CAP Sleep (PB-CAPSD) database [12]. This particular subject experienced periodic limb movement disorder. The figure highlights distinct EEG behaviors associated with each sleep stage, annotated to showcase their waveform characteristics. The N1 stage represents a transitional phase between light and deep sleep. In this stage, the EEG predominantly contains alpha waveforms, constituting about 2–5% of total sleep. Moving to stage N2, waveforms such as sleep spindles and k-complexes are prevalent, covering approximately 40–60% of total sleep for one subject [12]. Finally, the REM stage behavior closely resembles the wake stage, featuring sawtooth waves with alpha and theta activities [13]. The interconnected changes in sleep behavior during transitions between stages play a vital role in studying mental and physical health. Individuals with various sleep disorders often deviate from a regular sleep cycle [14].

Therefore, classifying sleep stages, particularly N1 or an extended transition period like N2, is crucial for identifying irregularities during sleep. In routine practice, sleep experts traditionally manually record multiple EEG signals and label them with corresponding sleep stages, making the entire process labor-intensive, time-consuming, and costly [15].

In the intersection of brainwave analysis and machine learning, extracting features from EEG signals plays a pivotal role. Wavelet transform, for instance, can analyze signals at multiple scales, making it valuable for detecting episodic events or signal changes over time. This characteristic renders it suitable for identifying changes in EEG signals, such as sudden increases or decreases in activity, which may be associated with specific events. This suggests a promising avenue for leveraging machine learning techniques to enhance the accuracy of sleep pattern analysis.

Despite the successes seen with both single and multi-modal sleep staging methods, several notable drawbacks persist:

i) A generalized framework adaptable for the classification task from the conventional five-stage to two-stage sleep stages is lacking.
ii) Supervised classification models, while effective with known data, may struggle with new records and can misclassify significant sleep stage patterns. Additionally, the features extracted from these models may be limited and fail to capture the complexity of the original signals adequately.
iii) Misclassification of several epochs as belonging to either N1 or REM stages has been observed, directly impacting the accuracy performance of sleep staging algorithms.

This study aims to leverage multi-modal signal fusions and apply them using machine learning techniques to overcome the limitations of traditional methods in sleep scoring. The objective is to enhance the consistency of polysomnography scoring and develop classifiers with high accuracy for each sleep stage.

## Related research

Over the years, the researchers developed different sleep staging methods based on machine learning and deep learning techniques. Most studies can be categorized into i) single-channel-based and multi-channel-based methods. In [16] the authors analyzed the sleep characteristics epochs that were pooled, then screened the features and selected the most suitable features based on relevance. In [17], the authors employed a band-pass filter during pre-processing to eliminate artifacts from the data. Their method yielded superior outcomes compared to existing procedures. Specifically, their approach proved effective for detecting dishonesty in EEG-based Brain-Computer Interface (BCI) systems.

In [18], the authors employed an orthogonal convolutional neural network (OCNN) to extract features from recorded polysomnography signals. They conducted their analysis on two publicly available sleep datasets from UCD and MIT-BIH. The OCNN model achieved accuracies of 88.4% and 87.6% with the UCD and MIT-BIH datasets, respectively. In [19], the author employed multi-modal classification and decision-making systems for sleep staging, incorporating an external neural network. The experimental work utilized the CAP sleep dataset, and the results indicated that the model performed well compared to an individual CNN model. The proposed model achieved a high accuracy of 95.43% for the six-class classification problem. In [20], the author introduced a novel approach for automated scoring of different stages of sleep using EEG signals collected from a single channel. This method utilized a unique cascaded recurrent neural network (RNN) architecture. The EEG data underwent preprocessing 55 times, and frequency-domain features were extracted, with the most relevant features selected via feature reduction techniques. Overall, the model achieved a classification accuracy of 86.7% for the five stages of sleep. The primary focus of this effort was to improve classification performance in sleep stage N1, with the aim of achieving satisfactory results in the remaining sleep stages as well. In reference [21], a novel method for automatic sleep stage categorization using EEG information from a single channel was proposed. The main idea is to directly apply the raw EEG signal to a deep convolutional neural network (CNN), bypassing the traditional feature extraction and selection process used in previous approaches. The suggested network architecture consists of nine convolutional layers followed by two fully connected layers. The proposed method achieved an accuracy above 90% for categorizing two to six classes, representing an improvement over existing methods. Additionally, Cohen's Kappa coefficients were reported as 0.98, 0.94, 0.90, 0.86, and 0.89, respectively, indicating strong agreement between predicted and actual sleep stages. In [22], the author utilized the concept of a weighted undirected network by mapping the feature vector into it. This network's various structural and spectral characteristics were separated. In [23] the author used multi-scale deep neural architectures, in which the decomposed signals were input into the CNN model for further analysis of the sleep patterns. The model resulted in an accuracy of 80.7% using S-EDF and 86.5% with the MASS dataset. In [24], the author used semi-supervised learning techniques for a better presentation of EEG signals for sleep staging. The author used two public datasets for this research work. The model received accuracy of 70.01% and 50.36% with S-EDF and ISRUC-Sleep datasets respectively. In [25], the author introduced a lightweight automated sleep staging system designed specifically for children, utilizing a single-channel EEG signal. The author combined Convolutional Neural Network (CNN) and Long Short-Term Memory (LSTM) models for classifying sleep stages. The experiments were conducted using two datasets: a children's sleep dataset and the Sleep-EDFx dataset. The system achieved an accuracy of 83.06% with the children's sleep dataset using the F4-M1 channel and 86.41% with the Sleep-EDFx dataset with manual feature extraction. In [26], the authors used multi-branch one-dimensional convolutional neural networks and extracted different frequency domain features from single-channel EEG data. The model resulted from 90.31% accuracy, 95.30% specificity, and 65.73% F1score. In reference [26], the authors employed multi-branch one-dimensional convolutional neural networks (CNNs) and extracted various frequency domain features and achieved an accuracy of 90.31%, specificity of 95.30%, and an F1 score of 65.73%. Some of the recent studies on sleep staging are presented in Table 1.

**Table 1:** Recent research works carried out on automated sleep stage classification using EEG and PSG signals

Study and Year	Techniques	Classifier	Signal	Dataset	Classification Levels	Results (%)
Micheal Dutt 2022 [1]	Deep learning	CNN-CRF	EEG	sleep-EDF	Five	86.8%
Q. Shen 2023 [5]		Asymmetric Siamese neural network (ASNN)	EEG	Sleep-EDF-20, Sleep-EDF-78, SVUH-UCD		86.0%
						82.3%
						76.3%
Fan, J 2021 [27]		Two-scale CNN	EOG	MASS	Five	81.2%
				Sleep-EDF		76.3%
Yan, Rui 2019 [28]	Multi-modal Fusions	RF	EEG + EOG + EMG + ECG	CAP Sleep Database	Five	86.24%
Ghimatgar, Hojat 2019 [29]	Autoregressive (AR) coefficients	RF + Hidden Markov Model (HMM)	EEG	DREAMS Subjects	Five-Two	77.01%
						79.12%
						86.04%
						95.47%
Fernandez-Bla nco, Enrique 2019 [30]	CNN	1D-CNN	EEG	S-EDF [Expanded]	Five	92.66%
Shen, Huaming 2020 [31]	Improved model-based essence features	Bagged Trees	EEG	Dreams Subjects	Five-Two	79.90% 82.08% 88.22% 96.48%
				ISRUC database		81.65% 84.68% 90.54% 96.18%
				S-EDF [Expanded]		89.54% 90.98% 92.33% 94.34% 97.62%
Cooray, Navin 2019 [32]	Multivariate pattern analysis	RF	EEG EOG EMG	Montreal Archive of Sleep Studies (MASS)	Five	92%
Sun Chenglu 2019 [33]	Hierarchical sequential neural network	recurrent neural network (RNN)	EOG R-R interval (RR) signals	MASS	Five	84.4%
				Sleep Apnea		74.3%
Guillot, Antoine 2020 [34]	SimpleSleepNet	RNN	EEG EOG EMG	Dreem Open Dataset—Healthy	Five	89.9%
				DOD-O (Dream Open Dataset—Obstructive)		88.7%
Korkalainen, Henri 2020 [35]	Deep learning-based approach	RNN	EEG	S-EDF	Five	83.7%
			EEG + EOG			83.9%
						76.3%

This research proposed a multi-modal machine learning model aimed at identifying changes in characteristics across individual sleep stages during sleep hours. The model achieves this by fusing multi-modal signals to classify sleep patterns [36]. It has been observed that the EEG signal is the most effective for robust sleep staging analysis. However, accurately analyzing changes in sleep behavior across individual sleep stages remains challenging [37]. The EMG and EOG signals can be acquired and recorded relatively easily, and there is evidence demonstrating a correlation between EEG, EMG, and EOG signals during sleep [38, 39]. The objective is to enhance the consistency in polysomnography scoring and to develop classifiers with high accuracy for each stage of sleep. Recognizing the substantial influence that various sleep stages exert on arousal, our research seeks to address the gap in existing studies by investigating different irregularities [40].

The notable advancements made by this research investigation are summarized as follows:

- Development of an automated sleep staging system by integrating three modalities of polysomnography signals.
- Incorporation of subject-specific features, such as age, to enhance sleep staging performance. This addresses a gap in existing sleep studies, which often rely solely on traditional feature-based analyses without considering subject characteristics.
- Introduction of an efficient feature selection method, the ReliefF feature selection algorithm is employed to simplify the feature selection process.
- Employing the AdaBoost algorithm with a random forest as a base classifier for sleep stage classification. This approach improves model prediction accuracy and resilience to overfitting and missing data issues.
- Reduction of reliance on prior knowledge in the feature extraction stage through the introduction of efficient adaptive signal analysis techniques.
- Comprehensive representation of differences between sleep stages by leveraging multimodal sleep data.
- Evaluation of the impact of different feature selectors and classifiers on the classification performance. Testing of the proposed methodology with heterogeneous signal data, demonstrating high classification accuracy in discriminating between sleep stages associated with heterogeneous signal characteristics.

The complete research study is presented in six sections. In the first section, the importance of sleep is briefly discussed. The second section presented related studies on sleep staging. The third section briefly presented the proposed methodology. The fourth section illustrates experimental and simulation result analysis. The fifth section delves into the results obtained and compares them with existing relevant research contributions. Finally, the last section concludes with this research work.

## Materials and methodology

**Fig. 2:**
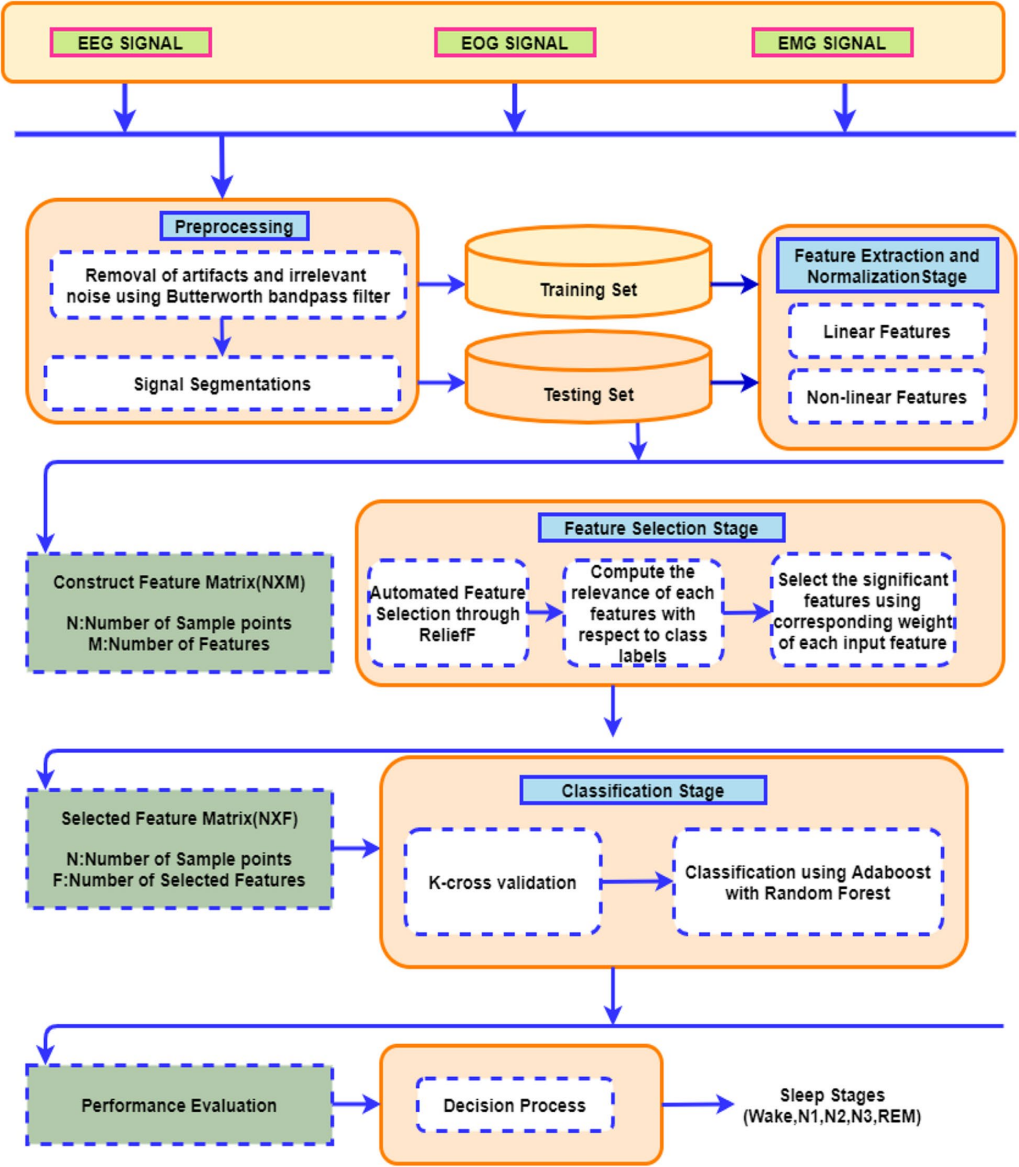
The complete layout of the proposed research work

In our investigation, we have combined AdaBoost with a foundational classifier called Random Forest (RF) for the classification of sleep stages. RF enhances the model's prediction variance by utilizing bootstrap sampling and selecting features via the ReliefF feature selection algorithm. Meanwhile, AdaBoost addresses the model's prediction bias by optimizing residuals. Consequently, by leveraging the strengths of these two algorithms, our research aims to enhance the performance of sleep staging in the model. This research also investigates the impact of age on sleep behavior, a factor often overlooked in recent studies. However, it's been noted that there exists a direct correlation between sleep patterns and the age of the subject. This insight is crucial for understanding variations in sleep characteristics across different stages. Recent contributions in sleep stage analysis have been critiqued for overlooking crucial aspects. For instance, they often neglect to consider the age factor when analyzing sleep behavior and fail to address imbalances in sleep epochs across different stages. Additionally, many studies overlook infrequent sleep stage transitions, such as subjects transitioning directly from wakefulness to deep sleep, particularly among healthy individuals [41]. The model is developed using multi-modal PSG signals. The complete framework of this research work is explained in Fig. 2.

### Sleep stages classes

According to the sleep rules established by R&K (Rechtschaffen and Kales) and AASM (American Academy of Sleep Medicine), sleep stages can be classified into two to six distinct classes. Details of sleep stage classification problems considered in this study are shown in Table 2.

**Table 2:** Description of sleep stages with AASM guidelines for this proposed work

Sleep Classes	5-Classes(5C)	4-Classes(4C)	3-Classes(3C)	2-Classes (2C)
Stages	REM vs. N3 vs. N2 vs. N1 vs. WAKE	REM vs. N3 N1 + N2 vs. WAKE	REM vs. NREM (N1 + N2, + N3) vs. WAKE	NREM + REM vs. WAKE

### Data description

#### ISRUC-Sleep subgroup1 database (ISRUC-SG1)

In this study, ISRUC-Sleep datasets were used, comprising sleep recordings from subjects having distinct medical conditions and affected by various types of sleep issues. These recordings were collected at the Hospital of Coimbra University from 200 to 2013 [41]. In the present work, 18 subjects were considered, among which 15 are male subjects and 4 female subjects, having an age range between 22–76 years.

#### Sleep-EDF database (S-EDF)

A whole of 8 Caucasian subjects' sleep recordings were collected. The collected recordings are mainly categorized into SC* and ST*. The SC* contained four subject recordings from healthy subjects. The ST* categories had four subjects with mild sleep problems. One EEG signal (Fpz-Cz), one EOG, and one EMG signal were recorded for category subjects [42].

#### Physio Bank CAP Sleep (PB-CAPSD) database

This dataset contained 108 polysomnographic recordings (CAP Sleep Database) [43]. This dataset collected EEG, EOG, EMG channels, and other electrophysiological signals. The detailed descriptions of this dataset were given in [43]. This research work retrieved PSG signals from six healthy subjects aged 23 to 37 years. The average period of sleep time for each subject is 8.5. The entire overnight polysomnography recordings were processed under the R&K rules. The number of subject recordings present in a particular dataset as classified into different sleep stages is presented in Table 3 below.

**Table 3:** Description of distribution of sleep stages

Database	ISRUC-Sleep Subgroup1 (ISRUC-SG1)	Sleep-EDF(S-EDF)	PhysioBank CAP Sleep Database	Sleep-EDF(S-EDF-78)
Number of Subjects	18	08	06	25
Gender (M/F)	15/04	04/04	04/02	14/09
Patient Age (years)	22–76	21–35	23–37	28–68
Epoch (seconds)	30 s	30 s	30 s	30 s
EEG Montage	Bipolar	Bipolar	Bipolar	Bipolar
Channel	EEG:C3-A2 EOG: ROC-A1 EMG: chin EMG (X1)	EEG:C3-A2 EOG: ROC-A1 EMG: chin EMG (X1)	EEG:C3-A2 EOG: ROC-A1 EMG: chin EMG (X1)	EEG:C3-A2 EOG: ROC-A1 EMG: chin EMG (X1)
Sampling Frequency (Hz.)	100 Hz	100 Hz	100 Hz	100 Hz
**Sleep Stages**				
Wake(W)	5103	8006(53%)	449(7%)	65,951(30%)
NREM-N1	2083	604	1405	21,522
NREM-N2	4346	3621	280	69,132
NREM-N3	2909	672	2162	13,039
REM	1767	1609	1751	25,835
Total Epochs	16,266	15,139	6047	195,479

#### Sleep-EDF-78 dataset

Sleep-EDF-78 is an expanded version of Sleep-EDF-20, comprising 197 overnight polysomnography (PSG) recordings. It includes annotated sleep stage information from 20 healthy subjects and 58 subjects experiencing mild sleep difficulties. The subjects range in age from 25 to 101 years, with 41 male and 37 female participants. These recordings feature various physiological signals, including EEG, EOG, and EMG [44].

**Fig. 3:**
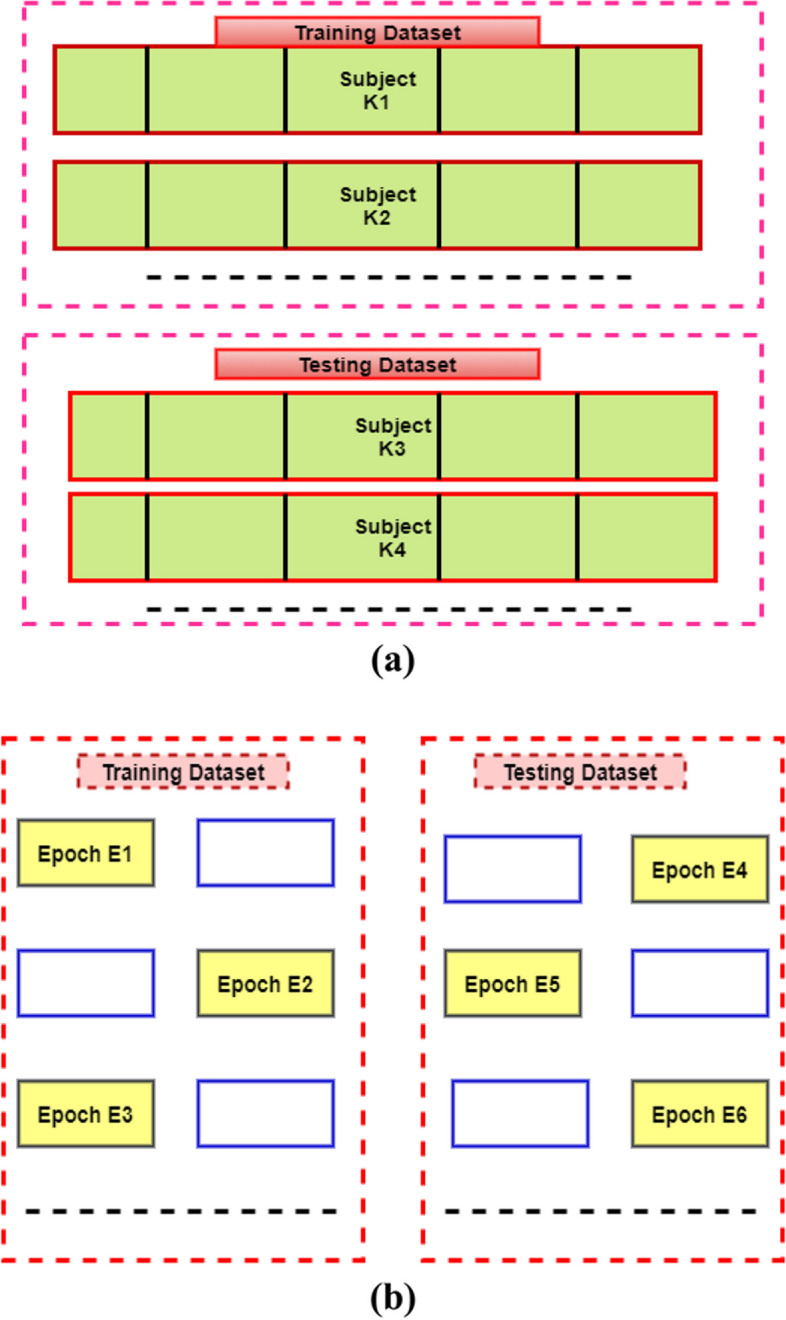
Training and Testing data partitioning methods: **a** subject-wise, **b** epoch-wise

**Fig. 4:**
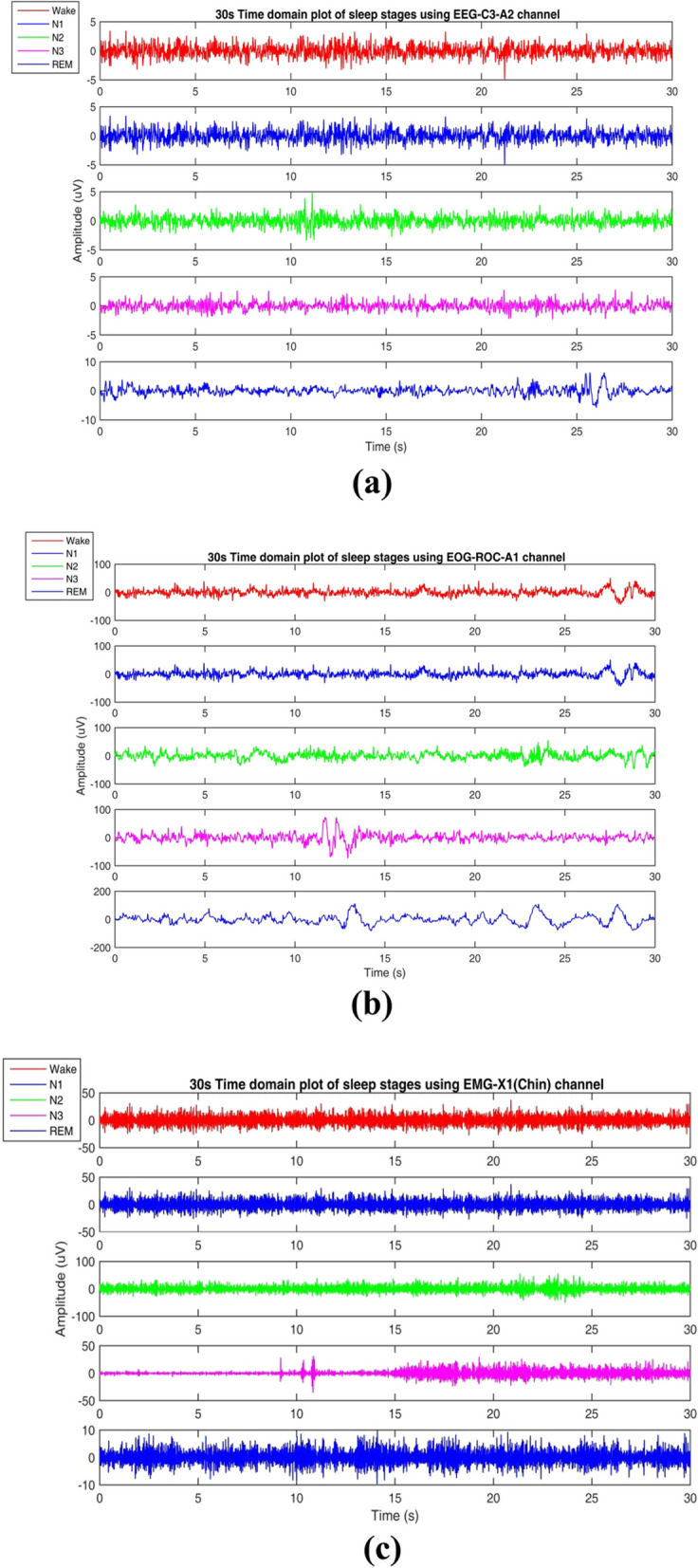
Sample recordings of the sleep-disordered subject-5 **a** using EEG signal **b** using EOG signal **c** using EMG signal of from ISRUC-Sleep dataset

**Fig. 5:**
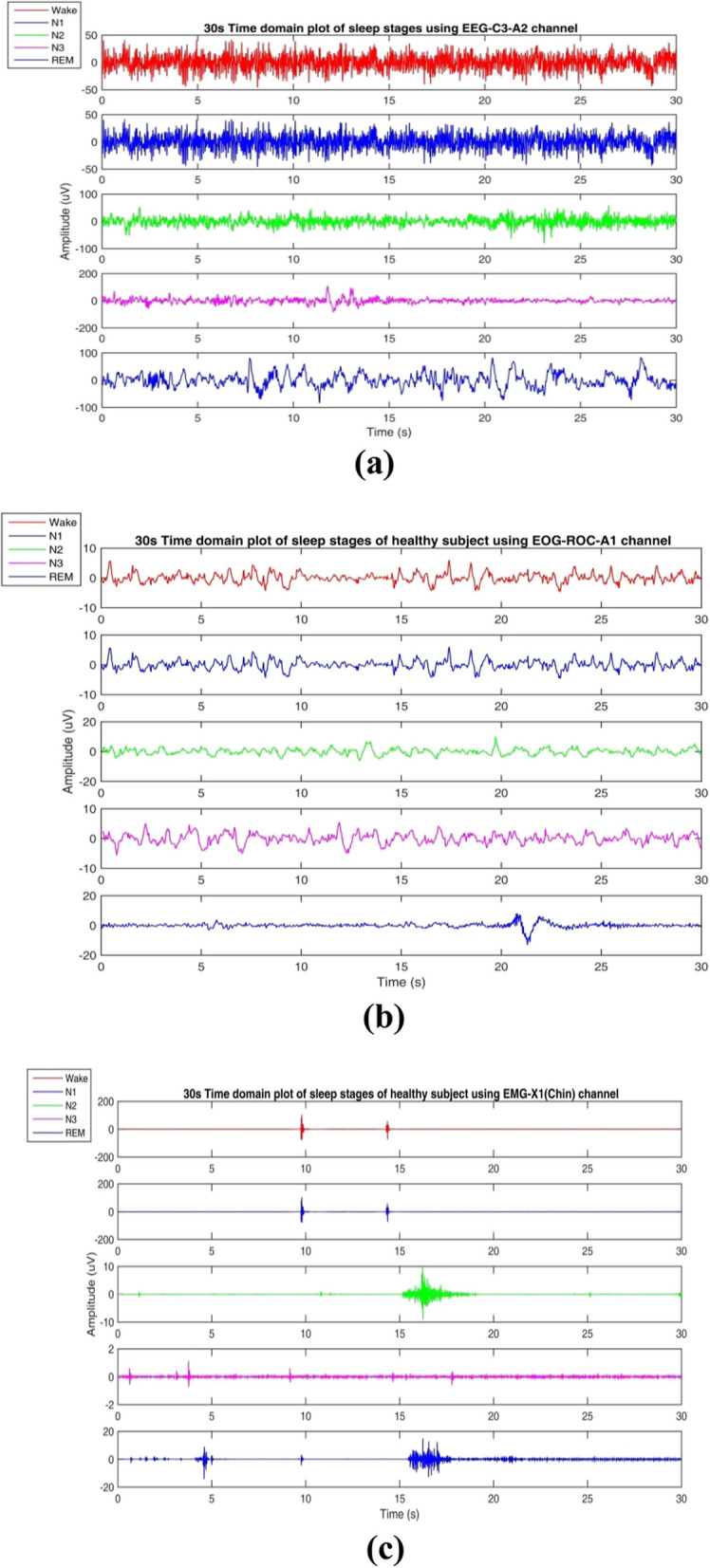
Sample Sleep recordings of the healthy controlled subject- sc4002e0 **a** using EEG signal **b** using EOG signal **c** using EMG signal of subject-1 from CAP Sleep Database

Generally, two different types of methods are more popular concerning clinical data; that is subject-wise (Subject-Independent Test) and epoch-wise (Subject-Dependent Test) (Fig. 3). This article uses the subject-wise and epoch-wise analysis methods on the ISRUC-SG1, S-EDF, and PB-CAPSD databases. Figure 4a-c presents the PSG signals recorded from the ISRUC-Sleep dataset of subject-5 with the 30 s of each sleep stage, including Wake, N1, N2, N3, and REM stages recorded on a subject affected by a small airway obstruction syndrome. In this case, the subject sleep cycle is continuously disturbed, and finds brief arousals in sleep, which causes the deprivation of REM and N3 sleep. Similarly, Fig. 5a-c presents the subject's sleep stages behavior recorded from the Sleep-EDF dataset of subject- sc4002e0, which was wholly healthy and controlled, with no sleep problems in earlier days.

### Preprocessing

In this research, artifacts were eliminated by employing a 10th-order Butterworth bandpass filter spanning frequencies from 0.5 to 49.5 Hz [45]. Generally, the raw signals are highly contaminated with different artifacts and irrelevant noises, which is difficult to process directly. To eliminate noise and artifacts, notch filtering, a high-pass filter with a cut-off frequency of 0.3 Hz, and a low-pass filter with a cut-off frequency of 30 Hz were applied to the EEG and EOG signals [46, 47]. To process the EMG signal, notch filtering, a high-pass filter with a cutoff frequency of 10 Hz, and a low-pass filter with a cutoff frequency of 75 Hz were utilized [48, 49]. All the preprocessing is performed through the MATLAB signal processing toolbox using digital filtering techniques.

### Features extraction

Feature analysis is of utmost importance to analyze subjects’ behavior to determine the parameters that significantly decide the classified stage [50]. The characteristics during sleep that are strongly correlated to the location that a particular epoch of sleep duration belongs to can be observed through feature extraction, which becomes even more necessary when the signals are highly random and unstable, as is the case with polysomnography signals [51–53]. The obtained features from different physiological signals are presented in Tables 4, 5, and 6, respectively.

**Table 4:** Extracted features from EEG Signal

Sl.No	Feature	Sl.No	Feature
1	Min_Value (MinV)	10	Hjorth Complexity (HC)
2	Max_Value (MaxV)	11	75th Percentile (75^th^P)
3	Std_Deviation (SD)	12	Skewness (SK)
4	Variance (VAR)	13	Kurtosis (KU)
5	Mean(M)	14	Zero crossing rate (ZCR)
6	Median (ME)	15–18	Relative Spectral Power
7	Mode (MO)	19–22	Band Power
8	Hjorth Activity (HA)	23–29	Power Ratios
9	Hjorth Mobility (HM)	30	Spectral Entropy (SE)

**Table 5:** Extracted features from EOG signal

Sl.No	Feature	Sl.No	Feature
31	Minimum Value (MinV)	39	Hjorth Mobility (HM)
32	Standard Deviation (SD)	40	Hjorth Complexity (HC)
33	Variance (VAR)	41	Skewness (SK)
34	Mean(M)	42	Kurtosis (KU)
35	Median (ME)	43	Zero crossing Rate (ZCR)
36	Mode (MO)	44	Permutation Entropy (PE)
37	Hjorth Activity (HA)	45	Spectral Entropy (SE)
38	Power Spectral Density (PSD)	46	Hurst Exponent (HE)

**Table 6:** Extracted features from EMG signal

Sl.No	Feature	Sl.No	Feature
47	Minimum Value (MinV)	55	Hjorth Complexity (HC)
48	Standard Deviation (SD)	56	Skewness (SK)
49	Variance (VAR)	57	Kurtosis (KU)
50	Mean(M)	58	Zero crossing rate (ZCR)
51	Median (ME)	59	Permutation Entropy (PE)
52	Hjorth Activity (HA)	60	Spectral Entropy (SE)
53	Hjorth Mobility (HM)	61	Energy (EN)
54	Spectral Edge (SPE)	62	Approximate Entropy (AE)
63	Age		

### Feature normalization and reduction

#### Feature normalization

After the feature extraction, a feature set with the dimensions of 16266 × 63, 15139 × 63, and 6047 × 63 for multi-modal PSG signals using ISRUC-SG1, S-EDF, and PB-CAPSD, respectively. Generally, the subject's data for both the baseline and the time series information have different orders of magnitude. Train the ML-based classification model makes converging difficult [51, 54]. To confirm that every feature data has to be level of the same standards, feature values were standardized using the z-score method. Zero mean and unit variance have been used here, after which a normalized feature vector is generated. This, in general, boosts the system’s performance and helps to remove the outliers.

#### Feature reduction

It is also one of the critical steps during the sleep staging process. It has been found that sometimes improper signal fusions may degrade the model’s performance. For this reason, it's essential to screen the best convenient feature, which helps discriminate the parts based on their characteristic changes over the individual sleep stages [55]. This study employs the ReliefF (ReF), a supervised feature weighting algorithm, to extract relevant features. The extracted features and their corresponding weights are presented in Tables 7, 8, 9, and 10, respectively.

**Table 7:** EEG features with their ReliefF weights

Weightage Order	Feature No	Feature Name	Weight
1	30	SE	0.96
2	9	HM	0.87
3	14	ZCR	0.81
4	15	Pow_Ratio5	0.77
5	28	RSP_delta	0.61
6	24	beta_powbp	0.55
7	23	Pow_Ratio2	0.52
8	19	delta_powbp	0.48
9	22	Pow_Ratio1	0.45
10	27	alpha_powbp	0.44
11	29	Pow_Ratio7	0.42
12	17	Pow_Ratio6	0.39
13	16	RSP_alpha	0.36
14	21	RSP_theta	0.25
15	25	Pow_Ratio4	0.19
16	20	theta_powbp	0.11
17	3	SD	0.07
18	8	HA	0.05
19	4	VAR	0.04
20	11	75th P	0.04
21	12	SK	0.03
22	18	RSP_beta	0.02
23	1	MinV	0.02
24	13	KU	0.02
25	6	ME	0.01
26	5	M	0.01
27	26	Pow_Ratio4	0.01
28	10	HC	0.01
29	02	MaxV	0.01
30	07	Mode	0.01

**Table 8:** EOG features with their ReliefF weights

Weightage Order	Feature No	Feature Name	Weight
1	45	SE	0.96
2	38	PSD	0.90
3	46	HE	0.89
4	43	ZCR	0.87
5	39	HM	0.78
6	44	PE	0.77
7	35	ME	0.77
8	31	MinV	0.74
9	32	SD	0.73
10	33	VAR	0.70
11	37	HA	0.69
12	34	M	0.65
13	41	SK	0.64
14	42	KU	0.56
15	40	HC	0.34
16	36	MO	0.52

**Table 9:** EMG features with their ReliefF weights

Weightage Order	Feature No	Feature Name	Weight
1	54	SPE	0.96
2	60	SE	0.89
3	58	ZCR	0.82
4	62	AE	0.80
5	61	EN	0.79
6	51	ME	0.66
7	55	HC	0.48
8	50	M	0.48
9	53	HM	0.45
10	59	PE	0.44
11	48	SD	0.44
12	52	HA	0.36
13	56	SK	0.35
14	57	KU	0.32
15	49	VAR	0.29
16	47	MinV	0.27

**Table 10:** Features selected from EEG, EOG, and EMG signals fusions

EEG		EOG		EMG		EEG + EOG + EMG	
**Feature** **No**	**Feature** **Name**	**Feature No**	**Feature_** **Name**	**Feature No**	**Feature** **Name**	**Feature No**	**Feature** **Name**
30	SE	45	SE	54	SPE	30	SE
9	HM	38	PSD	60	SE	42	ZCR
14	ZCR	46	HE	58	ZCR	43	PE
15	RSP_delta	43	ZCR	62	AE	62	AE
28	Pow_Ratio6	39	HM	61	EN	38	PSD
24	Pow_Ratio2	44	PE	51	ME	55	HE
23	Pow_Ratio1	35	ME	55	HE	54	SPE
19	delta_powbp	31	MinV	50	M	61	EN
22	beta_powbp	32	SD	53	HM	28	RSP_delta
27	Pow_Ratio5	33	VAR	59	PE	39	HM
29	Pow_Ratio7	37	HA	48	SD	11	75th P
17	RSP_alpha	34	M	52	HA	37	HA

1–30: EEG Features; 31–46: EOG Features; 47–62: EMG Features

The AdaBoost meta-learning method is fed with the features from the ReF algorithm to produce base-learner random forest classifiers for accuracy improvement & mitigation of overfiring issues [56]. AdaBoost reinforces any base classification problem by boosting its accuracy. This approach is foolproof, simple, and convenient; it is rated much higher than its counterparts. Besides this, it has the added advantage of being non-parametric and performs much more reliably in figuring out the outlier information from training samples [57, 58]. One of the standout features of this algorithm is that it is agnostic of the presence of any weak learners. Hence it finds presence across many classification problems. The algorithm here is being fed with a training dataset TD which ranges over n sample values i.e. ${T}_{D}=\left({X}_{i},{Y}_{i}\right)$ for *i* = *1, 2,…N*; The variables ${X}_{i}$ & ${Y}_{i}$ = {0,1,2,3,5} represent feature vector and its labels respectively. The class labels 5,3,2,1 and 0 correspond to the REM, N3, N2, N1, and WAKE stages, respectively. Followed by this, the base level classification models is called over several times. The weak hypotheses are linearly combined to construct the final view at each round.

Random Forest (RF) is one of the most acceptable methodologies in classification, easily head-and-shoulders above its compatriots. It is one of the superiors among the various Bagging techniques [59]. The stand-out feature of this methodology is its ability to process massive datasets smartly and its capability to deal with large volumes of input variables without data loss and seamlessly characterizing the features of classification. Besides this, its ability to manage outliers and noise data are noteworthy. This algorithm is nothing but an aggregation of classifiers in an efficient tree structure. Each of the participating trees independently contains random sample values [60]. This is suitable for all other trees of the forest as well. The predictive results are derived using voting at each step, and subsequently, the highest voted predictive effect becomes the final prediction result.

Random Forest of AdaBoost algorithm is taken as the base classifier to classify the sleep stage. This duo has only ensured higher classification accuracy for all the sleep stages. The below algorithm presents their correlation as following Algorithm 3:

**Figure:**
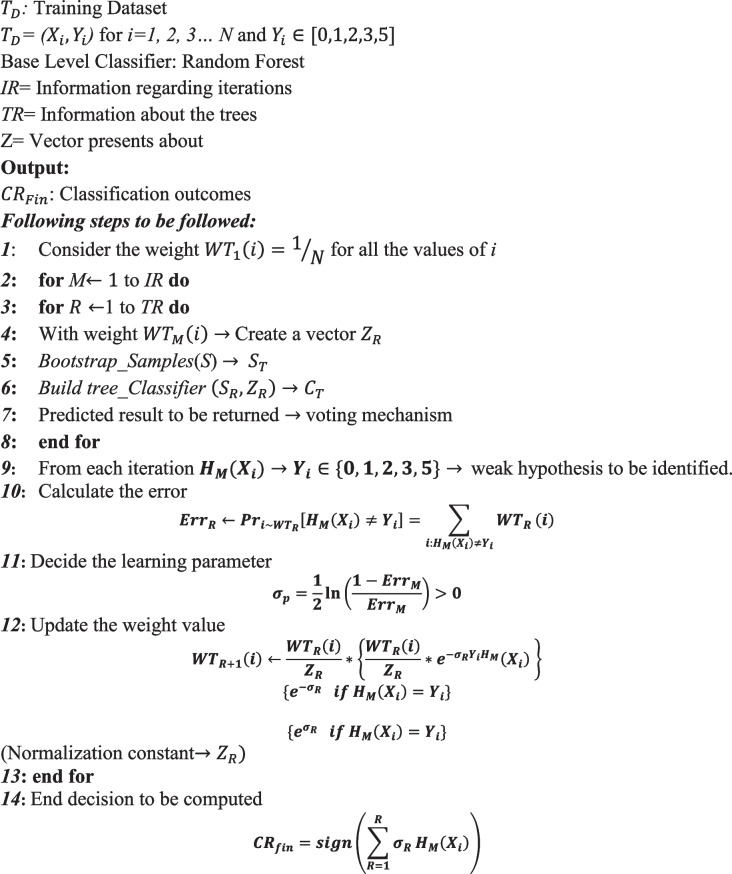
** Algorithm 3.** Random Forest of AdaBoost algorithm

### Experimental set-up

Only Polysomnography (PSG) signals have been considered for this work, which is a combination of the physiological signals pertaining to the three channels: EEG, EOG, and EMG. The entire duration of these recordings is segmented into epochs of 30 s. This study obtained two different testing schemes such as epoch-wise and subject-wise.The entire experiments were compiled and executed using MATLAB 2017b version.

#### Testing schemes

##### Epoch-wise Test (Subject Dependent Test)

In this testing scheme, tenfold cross-validation, considers all the samples to be mixed to evaluate the proposed model's performance. During this test procedure, both the training and testing samples were obtained from the same subject. So, the performance of this testing scheme may be overly optimistic and incomparable to the subject-wise analysis.

#### Subject-wise Test (Subject Independent Test)

During this testing method, we have obtained a cross-validation strategy to assign one set of data is considered as testing data while the others are treated as training datasets via 10-fold cross-validation. This testing procedure was repeated* K* times for *K* subjects. Each subject’s data is used to consider as the test in turn whereas other *K-1* subjects' data are considered for training the proposed classification model.

### Performance evaluation metrics

In this section, the performance of the model is measured using five different standard metrics such as accuracy (ACC) [61], sensitivity (SEN), specificity (SPC) [61], precision (PRE) [62], F1Score (F1Sc) [63], and Cohen's Kappa Score [64]. We used three public datasets such as ISRUC-SG1, S-EDF, and PB-CAPSDB datasets under AASM rules to assess the model's efficiency better.

This proposed study executes six individual experiments using multi-modal PSG signals based on two different testing schemes: epoch-wise and subject-wise. Table 10 presented the brief settings for all the experiments and all the experiments based on the two-class to five-class sleep stages classification. A total of 63 features were extracted, which includes 1–30 from EEG features, 31–46 from EOG features, and 47–62 from EMG features, respectively.

### Performance evaluation of proposed sleep staging model using an individual feature

To identify the impacts of the screened features of the EEG, EOG, and EMG signal for sleep staging, we investigate the individual features under the AASM sleep standards. The sleep staging performance will be analyzed on the basis of a single feature using the same datasets, testing schemes, and proposed classification model. We extracted 30 features from EEG signals (See Table 3), 16 features from EOG (See Table 4), and EMG (See Table 5) signals, respectively. Finally, the selection of suitable features based on the features' weight value signifies more suitability. The sorted features using the ReF feature selection algorithm for EEG, EOG, and EMG signals are presented in Tables 7, 8, and 9, respectively. The best top 12 extracted features from EEG, EOG, and EMG signals were presented in Table 10 for sleep staging. The selected features were fed one by one into AdaBoost with a base classifier as RF to evaluate the features' effectiveness with concern to discrimination of the sleep stages. The top best 12 selected features' accuracy performance during sleep staging for five sleep states classification was conducted in both the testing (epoch-wise and subject-wise) schemes. The same individual feature accuracy results were presented in Tables 11, 12, and 13 concerning EEG, EOG, and EMG signals, respectively.

**Table 11:** Overall accuracies of sleep staging using single-selected features with the Top 12 selected features of EEG signal

Feature Number	Selected Feature Names	Epoch-wise Testing (%)	Subject-wise Testing (%)
30	SE	72.79%	69.66%
9	HM	68.43%	64.32%
14	ZCR	62.38%	56.90%
15	RSP_delta	60.07%	51.38%
28	Pow_Ratio6	56.91%	47.79%
24	Pow_Ratio2	51.11%	43.09%
23	Pow_Ratio1	43.80%	39.60%
19	delta_powbp	43.91%	38.35%
22	beta_powbp	40.36%	36.77%
27	Pow_Ratio5	38.96%	35.65%
29	Pow_Ratio7	38.15%	35.12%
17	RSP_alpha	38.10%	34.76%

**Table 12:** Overall accuracies of sleep staging using single-selected features with Top 12 selected features of EOG signal

Feature Number	Selected Feature Names	Epoch-wise Testing (%)	Subject-wise Testing (%)
45	SE	74.79%	69.58%
38	PSD	68.43%	66.32%
46	HE	66.80%	56.60%
43	ZCR	61.27%	55.18%
39	HM	56.01%	49.79%
44	PE	47.11%	45.09%
35	ME	39.80%	37.60%
31	MinV	36.91%	35.05%
32	SD	33.10%	31.47%
33	VAR	29.96%	28.19%
37	HA	29.15%	25.12%
34	M	28.10%	25.12%

**Table 13:** Overall accuracies of sleep staging using single-selected features with the Top 12 selected features of EMG signal

Feature Number	Selected Feature Names	Epoch-wise Testing (%)	Subject-wise Training (%)
54	SPE	44.19%	40.76%
60	SE	38.33%	37.23%
58	ZCR	36.74%	34.16%
62	AE	36.56%	34.03%
61	EN	33.58%	31.44%
51	ME	32.94%	29.78%
55	HE	32.88%	29.11%
50	M	29.25%	27.35%
53	HM	28.87%	26.97%
59	PE	27.46%	26.09%
48	SD	26.84%	24.82%
52	HA	26.77%	24.02%

From Table 11, it has been observed that single EEG features using sleep staging are not performed well. The highest result achieved using the SE feature as 72.79%, and the lowest performance was reported as 38.10% using RSP_alpha based on epoch-wise testing scheme and similarly, the accuracy reported based on subject-wise for SE (69.66%) and RSP_alpha (34.76%) respectively.

It has been found from Table 12 that the same classification model reported the highest accuracy with the SE feature (74.79%) and lowest accuracy with the ME feature (28.10%) based on epoch-wise testing and similarly SE (69.58%) and M (25.12%) based on subject-wise testing using the top 12 selected features of the EOG signal.

From Table 13, it is noted that the sleep staging performance reached its peak with the SPE feature, achieving an accuracy of 44.19% and a sensitivity of 40.76% and lowest with the HA feature (26.77%) (24.02%) based on the epoch-wise and subject-wise testing schemes. Finally, it has been seen from Tables 10, 11, 12 that the performance of the sleep staging is relatively low since a single feature could only partially discriminate the sleep stages. Although single-channel and single-feature may experience this challenge during sleep staging, on the other hand, their combinations of the signals and features may better perform.

### Performance evaluation of the proposed sleep staging model using multi-modal signal fusions

In this section, the effectiveness of multi-modal signal fusions is analyzed during sleep staging. Six individual experiments are performed using three widely accepted public datasets as ISRUC-SG1, S-EDF, and PB-CAPSD. The brief experiment settings are presented in Table 14, and all the experiments are based on the classification of the five-sleep state. Experiment-1 to Experiment-3 use epoch-wise and Experiment-4 to Experiment-6 use subject-wise testing schemes, respectively. To compare the outcome of the proposed research work with the previous existing contributions on the ISRUC-SG1, S-EDF, and PB-CAPSDB, the experimental settings are set as similar as possible, which includes testing schemes dataset size, and input signals.

**Table 14:** Experiments under different testing procedures

Experiments	Datasets	Signals	Testing Schemes
Experiment-1	ISRUC-SG1	EEG + EOG + EMG	Epoch-wise
Experiment-2	S-EDF		
Experiment-3	PB-CAPSD		
Experiment-4	S-EDF-78		
Experiment-5	ISRUC-SG1	EEG + EOG + EMG	Subject-wise
Experiment-6	S-EDF		
Experiment-7	PB-CAPSD		
Experiment-8	S-EDF-78		

### Analysis of the sleep staging performance using Epoch-wise (Experiment-1 to Experiment-4)

For Experiment-1 and Experiment-4, different fusions of the signals were performed on three datasets such as ISRUC-SG1, S-EDF,PB-CAPSDB and S-EDF-78 under the AASM sleep scoring rules. The top 12 selected features are presented in Table 10. At last, the selected multi-modal features were fed into the ADB + RF classifier. The confusion matrices obtained using the epoch-wise testing scheme for Experiment-1 to Experiment-4 are detailed in Tables 15, 16, 17, 18. Furthermore, performance metrics for Experiment-1 to Experiment-4 are summarized in Tables 19, 20, 21.

**Table 15:** Confusion matrix for 5C sleep staging by ADB + RF classifier using EEG + EOG + EMG on ISRUC-SG1dataset

**Automatic Scoring**		**Expert Scoring**				
		**W**	**N1**	**N2**	**N3**	**REM**
	**W**	1144	2	5	4	19
	**N1**	5	675	13	10	12
	**N2**	11	30	1054	4	0
	**N3**	1	60	16	1112	36
	**REM**	11	22	12	5	617

**Table 16:** Confusion matrix for 5C sleep staging using EEG + EOG + EMG on S-EDF dataset

**Automatic Scoring**		**Expert Scoring**				
		**W**	**N1**	**N2**	**N3**	**REM**
	**W**	2378	33	6	1	5
	**N1**	15	113	5	1	25
	**N2**	2	28	1024	49	16
	**N3**	2	1	23	356	1
	**REM**	2	156	6	2	437

**Table 17:** Confusion matrix of EEG + EOG + EMG for 5C sleep staging with PB-CAPSDB dataset

**Automatic Scoring**		**Expert Scoring**				
		**W**	**N1**	**N2**	**N3**	**REM**
	**W**	404	3	7	4	8
	**N1**	16	291	3	4	12
	**N2**	21	9	455	4	8
	**N3**	2	2	7	224	5
	**REM**	6	4	6	8	302

**Table 18:** Confusion matrix of EEG + EOG + EMG for 5C sleep staging with S-EDF-78 dataset

**Automatic Scoring**		**Expert Scoring**				
		**W**	**N1**	**N2**	**N3**	**REM**
	**W**	62897	2018	689	71	476
	**N1**	622	11982	6221	112	2585
	**N2**	544	552	63646	2161	2229
	**N3**	16	7	1016	11988	12
	**REM**	902	2748	1158	92	20395

**Table 19:** Performance metrics values obtained for 5C sleep staging using an ISRUC-SG1 dataset with EEG + EOG + EMG

Testing Schemes	Sleep Stages	Accuracy	Sensitivity	Precision	F1-Score
**Epoch-wise**	**Wake**	98.76%	97.61%	97.44%	97.53%
	**N1**	96.76%	85.55%	94.41%	89.76%
	**N2**	98.54%	95.82%	95.91%	95.86%
	**N3**	97.13%	97.97%	90.78%	94.24%
	**REM**	97.52%	90.20%	92.50%	91.34%
**Overall**		**97.74%**	**93.43%**	**94.21%**	**93.75%**

**Table 20:** Performance metrics values obtained for 5C sleep staging using an S-EDF dataset with EEG + EOG + EMG

Testing Schemes	Sleep Stages	Accuracy	Sensitivity	Precision	F1-Score
**Epoch-wise**	**Wake**	98.49%	99.12%	98.14%	98.63%
	**N1**	94.23%	34.14%	71.07%	46.12%
	**N2**	96.68%	96.24%	91.51%	93.82%
	**N3**	98.18%	87.04%	92.95%	89.90%
	**REM**	95.29%	90.29%	72.47%	80.40%
**Overall**		**96.57%**	**81.37%**	**85.23%**	**81.77%**

**Table 21:** Performance metrics values obtained for 5C sleep staging using a PB-CAPSDB dataset with EEG + EOG + EMG

Testing Schemes	Sleep Stages	Accuracy	Sensitivity	Precision	F1-Score
**Epoch-wise**	**Wake**	96.16%	89.98%	94.84%	92.34%
	**N1**	96.93%	94.17%	89.26%	91.65%
	**N2**	96.43%	95.19%	91.55%	93.33%
	**N3**	97.90%	91.80%	93.33%	92.56%
	**REM**	96.71%	90.15%	92.64%	91.38%
**Overall**		**96.83%**	**92.26%**	**92.32%**	**92.25%**

From Tables 19, 20, 21, 22, it is evident that the average accuracy, sensitivity, precision, and F1 score are reported as 97.74%, 93.43%, 94.21%, and 93.75%, respectively, using the ISRUC-SG1 dataset, 96.57%, 81.37%, 85.23%, and 81.77% using S-EDF dataset, and 96.83%, 92.26%, 92.32% and 92.25% using PB-CAPSDB and 95.38%,80.25%,82.03% and 80.93% using S-EDF-78 respectively. But it has been seen that our proposed model is well-performed against the discrimination of N1 sleep stages. It is noticed in Tables 19 and 21 that the SEN-N1 sleep stage has improved using ISRUC-SG1 and PB-CAPSDB datasets, respectively. The classification performance results for the five-class (5C) to two-class (2C) using ISRUC-SG1 S-EDF, PB-CAPSDB and S-EDF-78 datasets based on epoch-wise testing schemes are presented in Table 23.

**Table 22:** Performance metrics values obtained for 5C sleep staging using a S-EDF-78 dataset with EEG + EOG + EMG

Testing Schemes	Sleep Stages	Accuracy	Sensitivity	Precision	F1-Score
**Epoch-wise**	**Wake**	96.97%	95.08%	96.79%	95.93%
	**N1**	92.00%	55.67%	69.23%	61.72%
	**N2**	95.57%	92.06%	96.31%	94.14%
	**N3**	98.00%	91.94%	83.11%	87.30%
	**REM**	94.37%	80.63%	79.37%	79.99%
**Overall**		**95.38%**	**80.25%**	**82.03%**	**80.93%**

**Table 23:** Performance of accuracy and Cohen's kappa score with top 12 selected features for ISRUC-SG1, S-EDF, and PB-CAPSDB scored according to AASM guidelines using epoch-wise testing procedures

Testing Schemes	Performance metrics	Dataset	Signals	5C	4C	3C	2C
				**(%)**	**(%)**	**(%)**	**(%)**
Epoch-wise	Overall Accuracy	ISRUC-SG1	EEG + EOG + EMG	94.30%	95.67%	97.21%	98.39%
		S-EDF		94.18%	95.09%	97.02%	98.10%
		PB-CAPSD		92.34%	94.89%	96.69%	97.79%
		S-EDF-78		95.38%	94.49%	97.01%	98.12%
	Cohen's kappa score	ISRUC-SG1	EEG + EOG + EMG	0.92	0.93	0.95	0.97
		S-EDF		0.90	0.91	0.93	0.97
		PB-CAPSD		0.90	0.91	0.92	0.96
		S-EDF-78		0.90	0.91	0.92	0.96

### Analysis of the sleep staging performance using Subject-wise (Experiment-5 to Experiment-8)

Here also we considered the same three public datasets for all the four experiments, the same multi-modal of signal features, and the only changes are here testing scheme that is subject-wise analysis. The other parameters remained the same as the earlier experiments of this study. The reported confusion matrix for Experiment-5 to Experiment-8 using ISRUC-SG1, S-EDF, PB-CAPSDB, S-EDF-78 based on subject-wise analysis are shown in Tables 24, 25, 26 and 27, respectively. Similarly, the proposed model's performance results based on the subject-wise testing procedure for all the datasets as mentioned earlier are presented in Tables 28, 29, 30, and 31 respectively. Finally, Table 32 presents the results for two-class (2C) to five-class (5C) sleep stages classification problems.

**Table 24:** Confusion matrix for Experiment 4 on ISRUC-SG1 using EEG + EOG + EMG for 5C sleep staging

**Automatic Scoring**		**Expert Scoring**				
		**W**	**N1**	**N2**	**N3**	**REM**
	**W**	2017	55	47	15	42
	**N1**	64	2788	66	107	55
	**N2**	35	27	4428	268	185
	**N3**	43	5	47	3043	11
	**REM**	62	117	143	9	2587

**Table 25:** Confusion matrix for Experiment 5 on S-EDF using EEG + EOG + EMG for 5C sleep staging

**Automatic Scoring**		**Expert Scoring**				
		**W**	**N1**	**N2**	**N3**	**REM**
	**W**	8002	103	43	44	55
	**N1**	26	543	22	13	3
	**N2**	392	55	2997	35	142
	**N3**	16	3	88	1173	19
	**REM**	122	107	53	8	1074

**Table 26:** Confusion matrix for Experiment 6 on PB-CAPSDB using EEG + EOG + EMG for 5C sleep staging

**Automatic Scoring**		**Expert Scoring**				
		**W**	**N1**	**N2**	**N3**	**REM**
	**W**	674	50	35	4	8
	**N1**	26	751	23	7	5
	**N2**	21	40	1625	64	88
	**N3**	2	2	37	1194	5
	**REM**	6	24	56	8	1292

**Table 27:** Confusion matrix for Experiment 6 on SEDF-78 using EEG + EOG + EMG for 5C sleep staging

**Automatic Scoring**		**Expert Scoring**				
		**W**	**N1**	**N2**	**N3**	**REM**
	**W**	62393	2518	691	74	475
	**N1**	1622	10982	6221	112	2585
	**N2**	544	1052	63146	2161	2229
	**N3**	16	7	1016	11988	12
	**REM**	902	2748	2158	92	19935

**Table 28:** Performance evaluation results for five-class sleep staging using ISRUC-SG1 with multi-modal signal fusions

Testing Schemes	Sleep Stages	Accuracy	Sensitivity	Precision	F1-Score
**Subject-wise**	**Wake**	97.62%	90.81%	92.69%	91.74%
	**N1**	96.77%	93.18%	90.52%	91.83%
	**N2**	94.21%	93.60%	89.58%	91.54%
	**N3**	96.71%	88.41%	96.63%	92.34%
	**REM**	95.97%	89.83%	88.66%	89.24%
**Overall**		**97.74%**	**93.84%**	**94.21%**	**93.95%**

**Table 29:** Performance evaluation results for five-class sleep staging using S-EDF with multi-modal signal fusions

Testing Schemes	Sleep Stages	Accuracy	Sensitivity	Precision	F1-Score
**Subject-wise**	**Wake**	94.51%	93.50%	97.03%	95.23%
	**N1**	97.65%	66.95%	89.46%	76.59%
	**N2**	95.01%	93.57%	82.77%	87.84%
	**N3**	98.39%	92.14%	90.30%	91.21%
	**REM**	96.44%	83.06%	78.74%	80.84%
**Overall**		**96.40%**	**85.85%**	**87.66%**	**86.34%**

**Table 30:** Performance evaluation results with multi-modal signal fusions for five-class sleep staging using PB-CAPSDB

Testing Schemes	Sleep Stages	Accuracy	Sensitivity	Precision	F1-Score
**Subject-wise**	**Wake**	97.33%	92.46%	87.42%	89.87%
	**N1**	96.90%	86.62%	92.49%	89.46%
	**N2**	94.97%	91.50%	88.41%	89.93%
	**N3**	97.77%	93.72%	96.29%	94.99%
	**REM**	96.46%	92.22%	93.22%	92.72%
**Overall**		**96.69%**	**91.30%**	**91.57%**	**91.39%**

**Table 31:** Performance evaluation results with multi-modal signal fusions for five-class sleep staging us-ing PB-CAPSDB

Testing Schemes	Sleep Stages	Accuracy	Sensitivity	Precision	F1-Score
**Subject-wise**	**Wake**	96.10%	94.32%	95.29%	94.80%
	**N1**	90.90%	51.03%	63.45%	56.57%
	**N2**	95.24%	91.34%	96.28%	93.75%
	**N3**	97.97%	91.94%	83.09%	87.29%
	**REM**	93.76%	77.16%	78.99%	78.07%
**Overall**		**94.79%**	**81.16%**	83.42%	**82.10%**

**Table 32:** Average overall accuracy and Cohen's kappa score with top 12 selected features for ISRUC-SG1, S-EDF, and PB-CAPSDB scored according to AASM guidelines using subject-wise testing procedures

Testing Schemes	Performance metrics	Dataset	Signals	5C	4C	3C	2C
				**(%)**	**(%)**	**(%)**	**(%)**
**Subject-wise**	**Overall** **Accuracy**	**ISRUC-SG1**	**EEG + EOG + EMG**	91.37%	94.42%	96.89%	98.23%
		**S-EDF**		91.08%	93.91%	96.19%	97.91%
		**PB-CAPSD**		91.55%	93.07%	95.91%	97.05%
		**S-EDF-78**		94.79%	95.05%	97.23%	98.10%
	**Cohen's kappa** **coefficient**	**ISRUC-SG1**	**EEG + EOG + EMG**	0.89	0.92	0.95	0.98
		**S-EDF**		0.87	0.91	0.94	0.97
		**PB-CAPSD**		0.89	0.91	0.92	0.97
		**S = EDF-78**		0.90	0.91	0.92	0.97

### Analysis of sleep staging classification performance using single-channel and multi-modal signals fusions

This analysis was done through the same three datasets in both the testing procedures (epoch-wise and subject-wise).

**Fig. 6:**
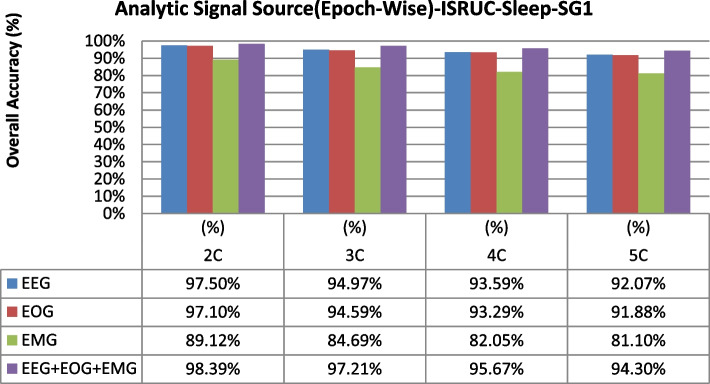
The overall accuracies performances of two-five sleep stages classification with ISRUC-SG1 dataset

**Fig. 7:**
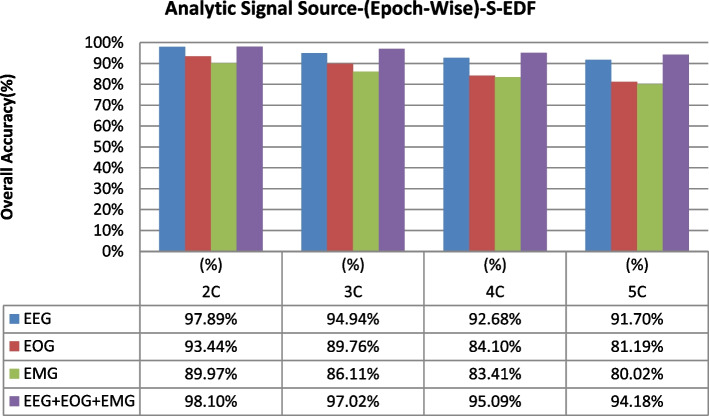
The overall accuracies performances of two-five sleep stages classification with the S-EDF dataset

**Fig. 8:**
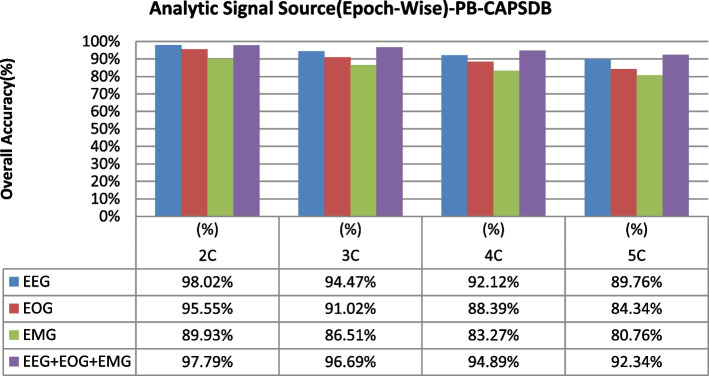
The overall accuracies performances of two-five sleep stages classification with ISRUC-SG1 dataset

**Fig. 9:**
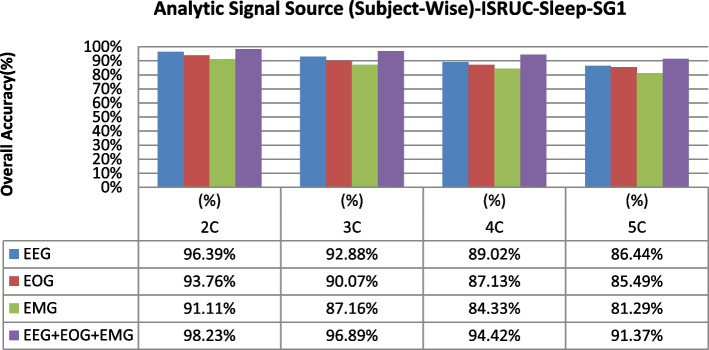
Overall accuracies performances of two-five sleep stages classification are compared between using single-channel and multi-modal of signals fusions using subject-wise testing procedures with ISRUC-SG1 dataset

**Fig. 10:**
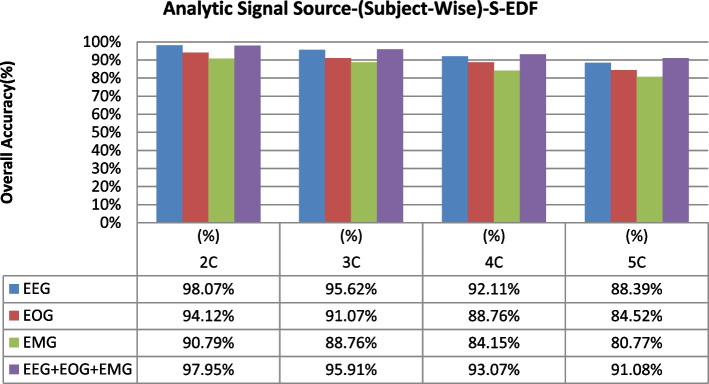
Overall accuracies performances of two-five sleep stages classification are compared in between using single-channel and multi-modal of signals fusions using subject-wise testing procedures with S-EDF dataset

**Fig. 11:**
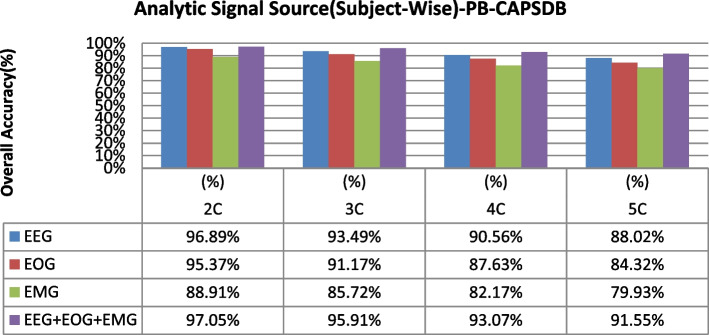
Overall accuracies performances of two-five sleep stages classification are compared between using single-channel and multi-modal signals fusions using subject-wise testing procedures with the PB-CAPSDB dataset

The overall accuracy performance for 2C to 5C classification problems using individual and multi-modal signal fusions using the epoch-wise testing method is shown in Figs. 6, 7, and 8. Similarly, the reported graph performance results using subject-wise testing procedures are shown in Figs. 9, 10, and 11 with ISRUC-SG1, S-EDF, and PB-CAPSDB, respectively. It has been noticed from the above presented graphical results that the overall accuracy performances are improved with combinations of the multi-modal signals incomparable to the individual signal performances with both the testing procedures irrespective of the obtained dataset in this study.

## Discussion

Several studies on sleep staging methods were generally focused upon the classification methods [16–40]. Some of the sleep studies were based on traditional time–frequency analysis using machine learning techniques [20–33], and the deep learning techniques [34, 37, 39, 40, 51, 55–64]. Generally, during the sleep studies, several kinds of signals were recorded for analyzing the changes in sleep characteristics during sleep. Generally, it is preferable and advantageous for considering the multi-modal of signal fusions during sleep quality assessment incomparable to the individual signals [65–79]. By the experiments results using both the testing schemes (epoch-wise and subject-wise), it is concluded that the multi-modal of signal fusions can be discriminating the sleep stages by AdaBoost with base classifier as RF in acceptable level. Therefore, this proposed methodology is much more effective than other machine learning models. The effectiveness of the multi-modal of signals using epoch-wise and subject-wise testing during sleep staging was illustrated in Tables 23 and 32, respectively. The sleep staging accuracy performances for two-five sleep classes' problems using individual and multi-modal signal fusions were illustrated in Figs. 6, 7, 8 and Figs. 9, 10, 11 based on epoch-wise and subject-wise testing, respectively. More specifically, the proposed multi-modal signal fusions (EEG + EOG + EMG) contain valuable information regarding changes in sleep characteristics during sleep periods. EEG signals capture the brain's information and its activities during sleep, and it also helps to study the changes in rhythm (alpha, delta, theta, and beta) during the different sleep stages [80–83]. Similarly, the EOG recorded the eye movement information, recognizing the W and REM stages [84–86]. EMG signals obtained information about muscular activity, and it has been found that the higher muscular behavior seen during the W stage is incomparable to REM stages. This information helps to discriminate the W and REM stages properly. Therefore, three modalities of signal fusions (EEG + EOG + EMG) signals to help discriminate the NREM sleep stages (N1, N2, and N3) and extracted multi-modal signal features that support discriminating the sleep status in the various aspect, which directly contributes to the improvement on sleep staging accuracy [59–65]. The proposed study investigated 63 features (time-domain, frequency-domain, and non-linear features) from the polysomnography signals' three modalities (EEG, EOG, and EMG).The selected joint optimal features were applied to the proposed classification model (AdaBoost with RF). For measuring the proposed methodology's effectiveness, both the testing procedures (epoch-wise and subject-wise) were adopted in our experiments. The present study was performed on three widely used datasets such as ISRUC-SG1, S-EDF, and PB-CAPSDB to analyze the effectiveness of the proposed methodology.

**Fig. 12:**
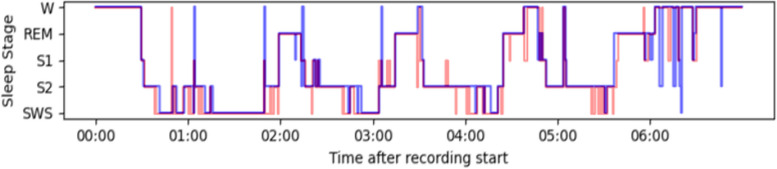
Comparison of hypnogram annotation of manual sleep staging (blue) and proposed sleep staging method (red) with ISRUC-SG1 dataset

**Fig. 13:**
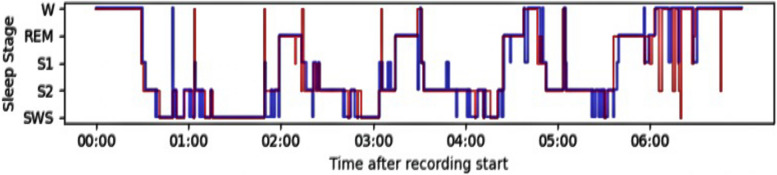
Comparison of hypnogram annotation of manual sleep staging (blue) and proposed sleep staging method (dark red) with S-EDF dataset

**Fig. 14:**
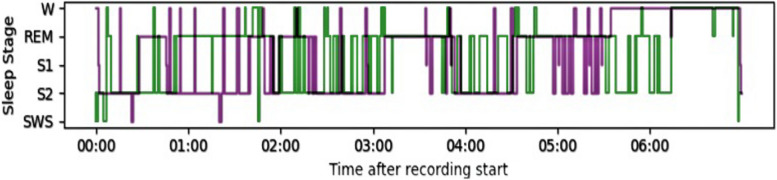
Comparison of hypnogram annotation of manual sleep staging (green) and proposed sleep staging method (purple) with PB-CAPSDB dataset

The required recordings were retrieved from the subjects who had difficulty sleeping and subjects with complete healthy control. To see the effectiveness of the sleep staging performance, the classification results provided by the proposed model(automatically) and manual staging are shown in Figs. 12, 13, and 14, where the hypnograms of ISRUC-Sleep-SG1, S-EDF, and PB-CAPSDB datasets are utilized.

However, there are some limitations to this study. Our study has certain constraints. Initially, we exclusively utilized physiological signals from PSG for sleep stage classification, yet sleep stages are also associated with additional features of the human body. Certain diseases can impact sleep stage classification. To enhance accuracy and provide a more comprehensive evaluation of sleep stages, we plan to extract information pertaining to sleep quality from electronic medical records in future research. Secondly, to address the challenge of minority classification in sleep stages, we will explore the implementation of data augmentation strategies to generate new sequences of sleep stages. Another limitation is that the utilization of a more varied array of machine learning models was not feasible for the experiments. Additionally, the optimization during the sleep stages phase was time-consuming due to the adoption of ReF, a method that systematically removes features one by one to confirm performance.

### Complexity comparison with other approaches

Many researchers have proposed two to five sleep state classification problems so far. For measuring the effectiveness of our proposed methodology during sleep staging, very brief comparisons were made with the existing state-of-the-art sleep staging methods with other state-of-the-art models. Figures 12, 13 and 14 illustrates the comparison between labels manually acquired by sleep experts and those predicted by the proposed method using one-night data records. Figures 12, 13 and 14 depicts the sleep data, revealing that the subject experienced approximately 6 h of effective sleep time. The individual entered a deep sleep phase shortly after initially falling asleep. Despite numerous instances of waking up during the sleep period, the subject promptly transitioned back into a sleep state after each awakening. We also compared the results with the latest published research works like LGSleepNet [5] SleepEEGNet [79], TinySleepNet [87], XSleepNet [88], CoSleepNet [89], SSleepNet [90], and RobustSleepNet [91] based on the multi-modal signal fusions and the same dataset. As observed in Tables 33, 34, and 35, our proposed sleep staging classification method has demonstrated superior performance compared to other methods across three datasets. From the comparison analysis, it has been found that the proposed multi-modal signal fusions performed high sleep staging classification accuracy. The reported overall accuracy and Cohen’s kappa score reported as 94.30%,0.92 (using ISRUC-SG1),94.18%,0.90 (using S-EDF), 92.34%,0.90 (using PB-CAPSDB) for five-class (5C) classification problem using epoch-wise analysis. Similarly, the same proposed model reported as 91.37%, 0.89 (using ISRUC-SG1), 91.08%, 0.87 (S-EDF), 91.55%, 0.89 (PB-CAPSD) using subject-wise analysis. Tables 23 and 32 demonstrate that the kappa score surpasses 0.80 using both testing procedures, indicating excellent agreement between manual and automatic scoring.Generally, in sleep staging, it's quite complicated and challenging towards discriminating between the N1 stage because it is the transition stage in between the Wake stage and the N2 stage. But it is noticed that the performance of the SEN-N1 stage is improved using subject-wise analysis with ISRUC-SG1 (93.18%), S-EDF (66.95%), and PB-CAPSDB (86.62%) incomparable to result reported using epoch-wise testing.

**Table 33:** Performance evaluation results in between proposed studies with state-of-the-art based upon the signals used

Reference/Year	Methods	Accuracy
Ref [31], 2018	EEG + EOG + EMG	80.07%
Ref [66], 2018		73.28%
Ref [67], 2019		92.09%
Ref [68], 2019		91.22%
Ref [84], 2022		90.60%
Ref [85], 2023		89%
Ref [86], 2023		90.21%
Ref [88], 2021 XSleepNet		81.1%
Ref [91], 2021 RobustSleepNet		78.2%
**Proposed** **Study** **(Epoch-wise** **Testing)**	**EEG + EOG + EMG + ** **AdaBoost with RF**	**94.30%**
		**94.18%**
		**92.34%**
**Proposed** **Study** **(Subject-wise** **Testing)**		**91.37%**
		**91.08%**
		**91.55%**

**Table 34:** Performance comparisons based on the dataset obtained for the experiment

Authors	Datasets	Epoch Number	Data Selection	Method	CT-5 (%)
Hassan et al. 2016 Ref [2]	S-EDF	15188	10-Fold	EMD + Ensemble methods	90.11%
Rahman, M. M 2017 Ref [22]	Sleep-EDF	15139	10-Fold	DWT + RusBoosting	91.13%
Hassan et al. 2017 Ref [63]	S-EDF	15188	10-Fold	EEMD + RUSBoost	83.49%
Zhou, J.et al. 2020 Ref [68]	S-EDF	15170	5-Fold	Stacked ensemble layer	91.8%
Zhou, J.et al. 2020 Ref [69]		15170	10-Fold	TLCNN-DF	93.58%
Satapathy, S. K. et al. 2021 Ref [70]		15139	5-Fold	Ensemble stacking model	91.10%
Satapathy, S. K. et al. 2021 Ref [71]		15,139	5-Fold	Ensemble	91.70%
Huang Zuo et al., 2022 Ref [72]		15188	10-Fold	Bagging Classifier	90.66%
Huafeng Wang et al., 2022 Ref [84]		15199	20-Fold	MSDNN + 1D-CNN	91.74%
Mousavi S, 2019 (SleepEEGNet) Ref [79]		42,308	10-Fold	Deep convolutional neural networks	84.26%
Weijia Yang et al., 023 Ref [87] TinySleepNet		44220	10-Fold	Deep Neural Model	85.4%
Xingfeng Lv et al., 2023 Ref [90] SSleepNet		42,308	10-Fold	Multi-scale feature extraction + Structured learning module	84.6%
**Proposed Study (Epoch-wise Testing)**	**ISRUC-SG1** **S-EDF** **PB-CAPSDB**	**16266** **15139** **6047**	**10-Fold**	**EEG + EOG + EMG + ** **AdaBoost with RF**	**94.30%** **94.18%** **92.34%**
**Proposed Study (Subject-wise Testing)**	**ISRUC-SG1** **S-EDF** **PB-CAPSDB**	**16266** **15139** **6047**	**10-Fold**	**EEG + EOG + EMG + ** **AdaBoost with RF**	**91.37%** **91.08%** **91.55%**

**Table 35:** Performance of ADB + RF model compared with the existing literature based on subject-dependent and subject-independent testing

Authors	Epoch Number	Validation	Classifier	CT-5 (%)
***Subject-Dependent Test (Epoch-wise Test)***				
Ref [26], 2019	127512	70%15%15%	1D-CNN	90.5%
Ref [31], 2020	104368	10-Fold	Bagged Trees	92.5%
Ref [73], 2020	62177	70%,10%,20%	CNN	83.3%
Ref [74], 2019	36972	10-Fold	HMM + RF	92.6%
Ref [75], 2021	1,139	20-Fold	TCNN + CRF	85.4%
Ref [76], 2017	106376	10-Fold	RF	91.5%
***Subject-Independent Test (Subject-wise Test)***				
Ref [29], 2019	40100	LOSO 50%-holdout	HMM + RF	81.2% 80.5%
Ref [68], 2020	42269	LOSO	ABNN + CNN	82.8%
Ref [77], 2021	81558	LOSO	RUSBoost	92.2%
Ref [78], 2017	41950	20-Fold	CNN + BLSTM	79.8%
Ref [79], 2019	42308	20-Fold	BiRNN + EDNA	82.8%
Ref [80], 2019	41950	LOSO	BLSTM + WDBN	85.5%
Ref [81], 2016	37022	20-Fold	CNN	74.8%
Ref [82], 2018	46236	LOSO	SVM-ARNN	82.5%
Ref [83], 2021	42308	20-Fold	HMM + CNN	84%
Ref [89], 2023 CoSleepNet	15139	10-Fold	Hybrid neural network architecture	87.11%
**Proposed Study** **(Epoch-wise Testing)**	**16266**	**10-Fold**	**EEG + EOG + EMG + AdaBoost with RF**	**94.30%**
	**15139**			**94.18%**
	**6047**			**92.34%**
**Proposed Study** **(Subject-wise Testing)**	**16266**	**10-Fold**	**EEG + EOG + EMG + AdaBoost with RF**	**91.37%**
	**15139**			**91.08%**
	**6047**			**91.55%**

### Computation time analysis

Another crucial factor for assessing a classifier is the computation time, although the training time is not taken into account during this analysis. During the initial phase, the computation time for each stage of the proposed ADB + RF method is logged, followed by the computation of the average value. The time consumption in seconds at various stages of the proposed scheme over Sleep-EDF is as follows: For each recorded signal, the computation time for feature extraction is 0.018 s, for feature reduction is 0.005 s, and for classification is 0.0013 s. The overall computation time for 3000 epochs is approximately 7 min, which is considered sufficiently fast to meet real-time requirements. Feature extraction is recognized to be more time-consuming, suggesting potential for optimization. Nevertheless, by utilizing only the top 12 features per epoch, not only are storage costs reduced, but calculations are also simplified and scheme surpasses other comparable methods in terms of feature usage and accuracy.

## Conclusion

Accurate and effective sleep staging is highly important step for analysis and identifying the sleep irregularities. To develop a highly accurate and robust automatic sleep staging system, this paper presents a computer-aided sleep staging system capable of classifying two to five sleep states using multimodal signal fusion of polysomnography (PSG) signals following the AASM sleep scoring guidelines. The proposed approach involves extracting multiple features, including time-based, frequency-based, statistical-based, entropy-based, and non-linear features, from three modalities (EEG, EOG, and EMG) of PSG signals. The model is evaluated on three widely accepted datasets: ISRUC-SG1, S-EDF, and PB-CAPSDB. These datasets include sleep recordings from subjects affected by various types of sleep-related disorders as well as healthy control subjects, totaling 16,266 epochs (ISRUC-SG1), 15,139 epochs (S-EDF), and 6,047 epochs (PB-CAPSDB) of 30-s length each. In this study, the chosen optimal features are inputted into highly robust, adaptable, and scalable classifiers such as AdaBoost with Random Forest as base classifiers. This approach directly contributes to enhancing classification accuracy. The entire experiments of this study were conducted through two testing procedures, epoch-wise and subject-wise. From the experimental results, it has been observed that the proposed methodology using multi-modal signal fusions is superior to other machine learning classification models with overall accuracies of 98.39%,97.21%,95.67%, and 94.30% using ISRUC-SG1, 98.10%, 97.02%, 95.09%, and 94.18% using S-EDF, 97.79%,96.69%,94.89% and 92.34% using PB-CAPSDB and 98.12%,97.01%,94.49% and 95.38% using S-EDF-78 for two-five classes respectively. Further, the proposed model reported accuracies of 98.23%, 96.89%, 94.42%, and 91.37% using ISRUC-SG1, 97.95%, 95.91%, 93.07% and 91.08% using S-EDF, 97.05%, 95.91%, 93.07%, and 91.55% using PB-CAPSDB and 98.10%,97.13%, 95.05%, and 94.79% using S-EDF-78 for two-five classes respectively. We will extend our proposed work by integrating with different physiological signals, which can help us to detect more than one different type of sleep disorder simultaneously.

## Data Availability

Authors utilized publicly available dataset i.e., ISRUC-Sleep subgroup1 database (ISRUC-SG1) https://sleeptight.isr.uc.pt/ISRUC_Sleep/ Sleep-EDF database (S-EDF) https://www.physionet.org/content/sleep-edfx/1.0.0/ Physio Bank CAP Sleep (PB-CAPSD) database https://physionet.org/content/capslpdb/1.0.0/
